# Recovery of Biventricular Function After Catheter Intervention or Surgery for Neonatal Coarctation of the Aorta

**DOI:** 10.1016/j.jacadv.2023.100326

**Published:** 2023-05-10

**Authors:** Klea Hysko, Dagmar Hohmann, Dmitry Bobylev, Alexander Horke, Harald Bertram, Christoph M. Happel, Georg Hansmann

**Affiliations:** a Department of Pediatric Cardiology and Critical Care, Hannover Medical School, Hannover, Germany; b Department of Cardiothoracic, Transplantation and Vascular Surgery, Hannover Medical School, Hannover, Germany

**Keywords:** 2D speckle tracking echocardiography, coarctation of the aorta, critical congenital heart disease, interventional cardiology, neonatal coarcation

## Abstract

**Background:**

Critical coarctation of the aorta (CoA) is a life-threatening condition in newborns that is associated with biventricular dysfunction.

**Objectives:**

The purpose of this study was to examine clinical outcome and echocardiographic changes in isthmus diameter and biventricular function in newborns with critical CoA treated with balloon dilation/stent placement or surgery.

**Methods:**

This is a retrospective single-center cohort study of 26 consecutive neonates with isolated critical CoA, who underwent transcatheter intervention (balloon angioplasty/stent; n = 10) or surgical CoA-repair (n = 16) (2012-2021). Isthmus dimensions and biventricular function at baseline and at hospital discharge were examined by echocardiography, including strain analysis of systolic and diastolic function using 2-dimensional speckle tracking.

**Results:**

Cardiogenic shock at hospital admission was more frequent in the interventional vs the surgical cohort (50% vs 25% of neonates). Echocardiographic isthmus diameter increased with therapy by 0.9 ± 0.1 mm and 1.0 ± 0.1 mm, respectively. Severe systolic left ventricular (LV) dysfunction was more common in interventional patients pre-therapy (LV ejection fraction <50% in 90% vs 38% of surgical patients), resulting in strongly reduced longitudinal strain (LV: −12.3% vs −16.3%; right ventricle:−13.8% vs −16.1% in the interventional and surgical patients, respectively). Prior to hospital discharge, all 26 patients had full recovery of biventricular systolic function, including normalization of longitudinal, radial, and circumferential LV strain and longitudinal right ventricular free wall strain. Improvement of LV diastolic function by strain analysis was evident in both cohorts pre-hospital discharge.

**Conclusions:**

Initial treatment of isolated CoA by percutaneous transcatheter intervention or surgical repair results in recovery of biventricular systolic function, making transcatheter treatment particularly suitable as rescue therapy for neonates with critical CoA.

Coarctation of the aorta (CoA) is a common congenital obstructive anomaly of the aortic arch, defined as a juxtaductal narrowing of the aortic isthmus. Critical CoA typically manifests in neonates after a sudden constriction or closure of the ductus arteriosus and is characterized by an imminent or actual cardiogenic shock. Transcatheter balloon dilation of the CoA ± stent placement are not routinely performed in neonates because of relatively high rates of restenosis and local complications such as femoral arterial injury and aortic aneurysms.^1^, ^2^, ^3^ There are, however, few reports on favorable outcomes in neonates with isolated coarctation presenting with cardiogenic shock/severe left ventricular (LV) dysfunction who underwent emergency surgical therapy.^4^ Other studies suggest that elective surgery unequivocally yields better intermediate and midterm results than transcatheter intervention.^5^^,^^6^ At our institution, percutaneous catheter intervention (balloon angioplasty ± stent) has been used as bridging therapy to definite surgery in neonates with critical isolated CoA. We hypothesized that both initial interventional and surgical approaches would successfully relieve LV pressure overload, leading to a recovery of systolic LV function prior to hospital discharge. Therefore, the goal of the study was to examine clinical outcomes and echocardiographic changes in isthmus diameter, biventricular structure and function in newborns with critical CoA treated with balloon dilation/stent placement or surgery. We also aimed to assess right ventricular (RV) function in neonates with isolated CoA, in order to determine the necessity of further comprehensive RV evaluation in follow-up examinations.

## Methods

### Patient population

**Figure 1:**
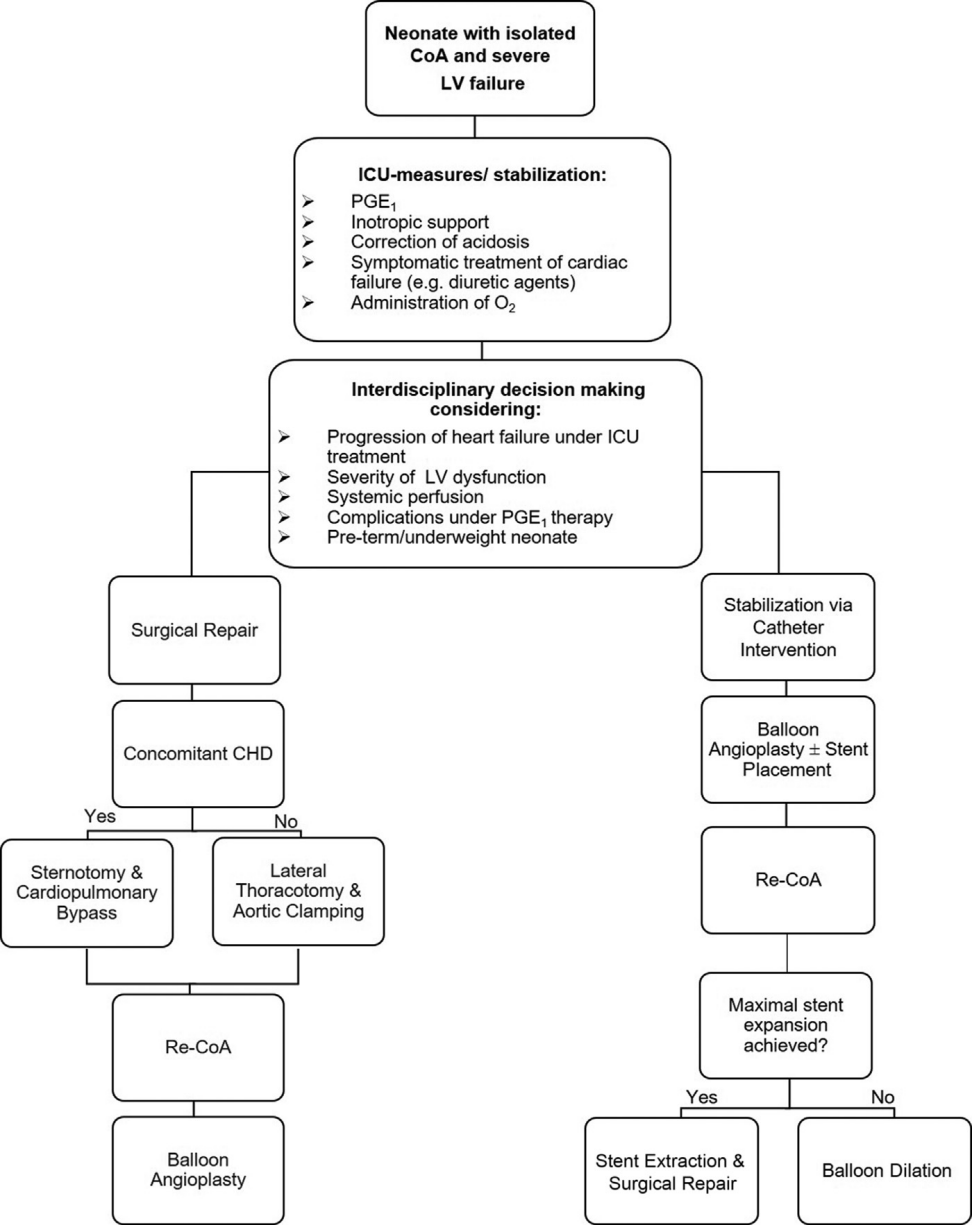
**Illustration of the CoA Treatment Algorithm at Hannover Medical School** Important decision-making involves an interdisciplinary team (including pediatric interventional cardiologists and congenital heart surgeons). Neonates with isolated, critical CoA often present with severe left ventricular dysfunction or even decompensated heart failure. Since surgical CoA repair is the therapeutic gold standard, patients at first need to be stabilized in the intensive care unit (ICU). Should they fail to do so or even deteriorate under ICU therapy, they undergo palliative transcatheter intervention of the CoA and eventually receive definitive elective surgical therapy. CHD = congenital heart defect; CoA = coarctation of the aorta; PGE_1_ = prostaglandin E_1_.

We conducted a retrospective cohort study of 26 consecutive neonates with isolated critical CoA, 10 of whom underwent balloon dilation ± stent placement, while the other 16 underwent surgical CoA repair at Hannover Medical School between December 2012 and May 2021. Supplemental Figure 1 shows an overview of our inclusion and exclusion criteria. Two patients of the interventional and 3 from the surgical cohort had small, hemodynamically insignificant atrial septal defects and were, therefore, not excluded from our study population. According to the treatment algorithm at our institution (Figure 1), the majority of critically ill patients receive catheter intervention while clinically stable patients primarily undergo surgical repair. Because of the aforementioned selection bias, we did not directly compare the 2 cohorts. Instead, we investigated the clinical course and cardiac function in the respective cohorts during the inpatient stay at our institution.

### Mode of treatment

Two of the interventional patients underwent only balloon angioplasty while further 8 patients received stent placement. The balloon diameter never exceeded the aortic diameter at the level of the diaphragm. Supplemental Table 1 provides detailed information on individual patients and procedure characteristics in the interventional cohort. Surgical patients underwent CoA resection or extended CoA resection, 11 of them via lateral thoracotomy and 5 under cardiopulmonary bypass via median sternotomy. One-half of the surgical patients (n = 8) received end-to-end anastomosis and the other half (n = 8) end-to-side anastomosis. The surgical procedures were performed by the same surgical team, with no essential changes in operative technique during the study period.

### Noninvasive imaging

All patients underwent advanced echocardiography pre- and post-therapy, ie, at hospital admission and shortly before hospital discharge. Echo-to-echo intervals were 10 ± 6 (median 9) days for the interventional patients and 11 ± 4 (median 10) days for the surgical patients. All examinations were performed on Philips IE33 or EPIQ 7 ultrasound machines by experienced cardiologists. The images were reviewed and reanalyzed using IntelliSpace Cardiovascular software (Philips Medical Systems).

We evaluated changes of the aortic isthmus (CoA site) focusing on the isthmus diameter as well as the isthmus/descending aorta diameter ratio, considering the descending aorta is a leading structure when calculating the envisaged isthmus diameter post-therapy. Maximal pressure gradient and maximal velocity (V max) across the isthmus by continuous wave Doppler (CW Doppler) were also used to quantify therapeutic success, thereby accounting for challenging Doppler interrogation and artifacts caused by the aortic stent in the interventional cohort. In M-Mode images of the parasternal short axis (PSAX) we assessed changes in LV and RV wall thickness (LV posterior wall thickness [LVPWD] and RV anterior wall thickness [RVAWD]), end-diastolic and end-systolic diameters (LV end diastolic dimension, LV end systolic dimension, and RV end-diastolic diameter [RVEDD]), and LV fractional shortening. In order to conventionally determine systolic LV performance, we also analyzed changes in aortic velocity time integral (AoV-VTI) by CW Doppler and LV ejection fraction (LVEF), by Simpson-monoplane method, in the apical 4-chamber (A4C) view as well as LV fractional area change (PSAX).

To evaluate cardiac remodeling, ventricular function and deformation pre- and post-therapy, offline strain analysis was performed using speckle-tracking software TomTec 2D CPA (2D Cardiac Performance Analysis, TomTec Imaging systems). The LV and RV longitudinal speckle tracking variables were measured in the A4C view (Supplemental Figure 2), while the radial and circumferential variables were assessed at the level of papillary muscle in PSAX. Except for the radial strain values, all other strain variables are negative, thus a decrease in strain values marks an improved LV or RV function. Systolic variables included the longitudinal, radial, and circumferential strain. We focused on the following diastolic variables: LV peak diastolic radial and circumferential strain rate and LV peak diastolic radial and circumferential velocity.

### Post-intervention follow-up

Eight of the interventional patients ultimately underwent surgical repair at our center. We analyzed clinical data and preoperative echocardiographic images (Supplemental Table 3). We also reviewed data of all interventional patients from their 1-year post-catheter intervention follow-up obtained by their pediatric cardiologists. A history of cardiac symptoms, assessment of femoral pulses (by palpation), and noninvasive arm/leg blood pressure differences were acquired from chart review.

### Statistical analysis

All measured data are presented as mean ± SEM and demographic data are presented as mean ± SD. Statistical analysis was based on echocardiographic data sets pre-and post-intervention and pre-and post-surgery. Normality was first assessed using the D’Agostino-Pearson and Shapiro-Wilk tests; data passed the tests, if *P* values were >0.05. Symmetrically-distributed data were then compared using the Student’s *t*-test, while the Wilcoxon paired 2-tailed *t*-test was performed on skewed data. *P* values <0.05 were considered significant. Bland-Altman plots were applied to assess intraobserver variability of strain variables (Supplemental Figure 3). All echocardiographic examinations were evaluated twice by the same observer within an interval of 2 months, primarily focusing on the LV longitudinal, radial, and circumferential strain as well as the RV free wall longitudinal strain. For the calculation of the intraclass correlation coefficient (ICC) we used measurements of all 26 patients cumulatively (2-way, agreement, single-score ICC, and calculated using the irr R package [ver.0.84.1]). Statistical analysis and creation of graphs were performed by using GraphPad Prism 6. More details on the selected variables can be found in the figure legends and in Supplemental Table 2.

### Ethics statement

All examinations were clinically indicated. Written, informed consent was obtained from the legal caregivers according to the principles expressed in the Declaration of Helsinki (IRB approval #10218, Ethics Committee, Hannover Medical School). All clinical data were anonymized.

## Results

### Demographic and clinical features at baseline

All demographic and clinical features of both cohorts are summarized in Table 1. Briefly, 80% of the interventional and 50% of the surgical patients were referred postnatally to our institution for further treatment. We noticed no significant differences in the demographic characteristics concerning age, height, weight, and body surface area at the time of therapy. There was one patient with Turner syndrome in the interventional cohort.

**Table 1:** Characteristics of Interventional and Surgical Patients

	Interventional Cohort (n = 10)	Surgical Cohort (n = 16)
Demographics		
Age at therapy (d)	14.6 ± 9.7	11.4 ± 4.4
Male	8 (80)	12 (75)
Weight (kg)	3.26 ± 0.7	3.31 ± 0.67
BSA (m^2^)	0.2 ± 0.025	0.2 ± 0.031
Preterm birth	2 (20)	3 (19)
Bicuspid aortic valve	3 (30)	7 (44)
Hypoplastic aortic arch	4 (40)	4 (25)
PFO/ASD II	2 (20)	3 (19)
Prenatal diagnosis	0 (0)	3 (19)
Clinical features		
NIBPGs at admission (mm Hg)	27.30 ± 12.82	27.7 ± 12.6
Femoral pulse		
Absent	4 (40)	6 (38)
Barely palpable	4 (40)	5 (31)
Strong	2 (20)	5 (31)
Decompensated heart failure	5 (50)	4 (25)
Progressive heart failure under ICU-therapy	6 (60)	0 (0)
Medical referral from other physicians and hospitals	8 (80)	8 (50)
CPR pre admission	2 (20)	0 (0)
NEC pre admission	1 (10)	1 (6.25)
Hospital stay (d)	13 ± 5	13 ± 4
Laboratory values at hospital admission (venous blood)		
Serum lactate (mmol/L)	5.78 ± 1.5	2.16 ± 0.25
pH	7.24 ± 0.06	7.38 ± 0.01
Base deficit (mmol/L)	−1.8 ± 1.8	−0.47 ± 0.73
Serum NTproBNP >35,000 ng/L (n/N measured, %)	4/7 (57)	2/9 (22)

Values are mean ± SD (for demographic data), n (%), or mean ± SEM (for laboratory values). Throughout the data, we notice no significant differences in demographic variables between our 2 cohorts. The interventional patients were however, more critically ill at hospital admission; more interventional patients presented with decompensated heart failure and more deteriorated under ICU therapy. One patient in either cohort had necrotizing enterocolitis prior to admission in our institution. Laboratory values also reflect worse metabolic derangement and worse acute cardiac distress in the interventional cohort.

AoV VTI = aortic valve velocity time integral; ASD II = Secundum atrial septal defect; BE = base excess; BSA = body surface area; CPR = cardiopulmonary resuscitation; ICU = Intensive care unit; n/n measured = number of patients in the cohort fulfilling the criteria out of the patients, in whom the variable was determined; NEC = necrotizing enterocolitis; NIBPGs = noninvasive systolic blood pressure gradient between right upper and lower extremity; NTproBNP = N-terminal pro-brain natriuretic peptide; PFO = patent foramen ovale.

While demographics were similar, the 2 cohorts were somewhat different in their clinical characteristics, 50% of the interventional patients and 25% of the surgical patients presented in cardiogenic shock at hospital admission at our institution. Six of 10 interventional patients further deteriorated despite intensive care therapy. One surgical and 8 interventional patients presented with metabolic acidosis on admission. Two of the interventional patients also required out-of-hospital cardiopulmonary resuscitation. In one patient resuscitative efforts were prolonged, with late return of spontaneous circulation, resulting in seizure activity later on.

### Nonresponders to PGE_1_ treatment frequently undergo catheter intervention

**Figure 2:**
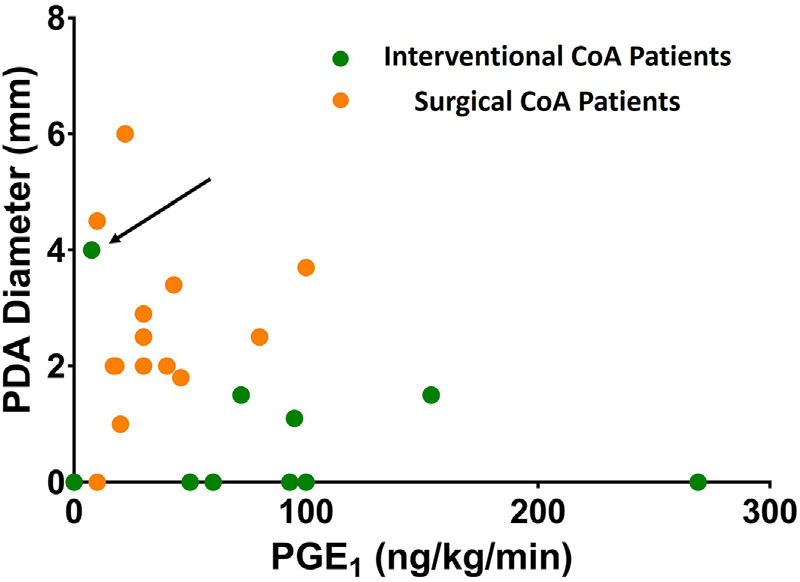
Poor Responders to PGE_1_ Commonly Undergo Transcatheter Intervention We correlated the maximal PGE_1_ dose the patients received, to the diameter of the patent ductus arteriosus (PDA) at that dose, measured in the suprasternal sagittal view, as a surrogate for PGE_1_ responsiveness of the PDA. Almost all interventional patients showed a weaker PGE_1_ responsiveness of the PDA despite administration of higher doses. One interventional patient **(arrow)** initially responded well to PGE_1_, but later developed necrotizing enterocolitis prior to admission at our center and was, therefore, considered too unstable for surgical repair of the coarctation of the aorta (CoA). One interventional patient was 28 days old at hospital admission, presented with a closed ductus arteriosus, and did not receive prostaglandin therapy. **Green** and **orange dots** represent individual interventional and surgical patients, respectively. PGE_1_ = prostaglandin E_1_.

No significant correlation between non-invasive systolic blood pressure gradients between right upper and lower extremity (NIBPGs) or maximal pressure gradient and the isthmus and patent ductus arteriosus (PDA) diameters (Supplemental Figures 4A to 4D) was observed. However, NIBPGs negatively correlated with LV performance independently of isthmus dimensions (Supplemental Figures 4E and 4F). We found the responsiveness of the PDA to PGE_1_ (prostaglandin E₁) to be more adequate and prognostically decisive than pressure gradients, when evaluating CoA progression and deciding on further CoA treatment in neonates. The PDA did not reopen in 6 of the interventional patients and only slightly increased in diameter in 2 others, despite administration of generally much higher PGE_1_ doses than in surgical patients (Figure 2), indicating unlikeliness of clinical stabilization in the intensive care unit. Only one of the interventional patients responded well to PGE_1_, but later developed necrotizing enterocolitis and underwent laparotomy and surgical resection of necrotic bowel prior to admission at our institution, and was thus considered too vulnerable for surgical CoA repair. One surgical patient developed necrotizing enterocolitis at an outside hospital as well but presented in stable condition after having received antibiotic treatment and supportive care.

### Both catheter intervention and surgical repair improve aortic isthmus diameter and maximal Doppler-blood flow velocity in neonatal CoA

**Figure 3:**
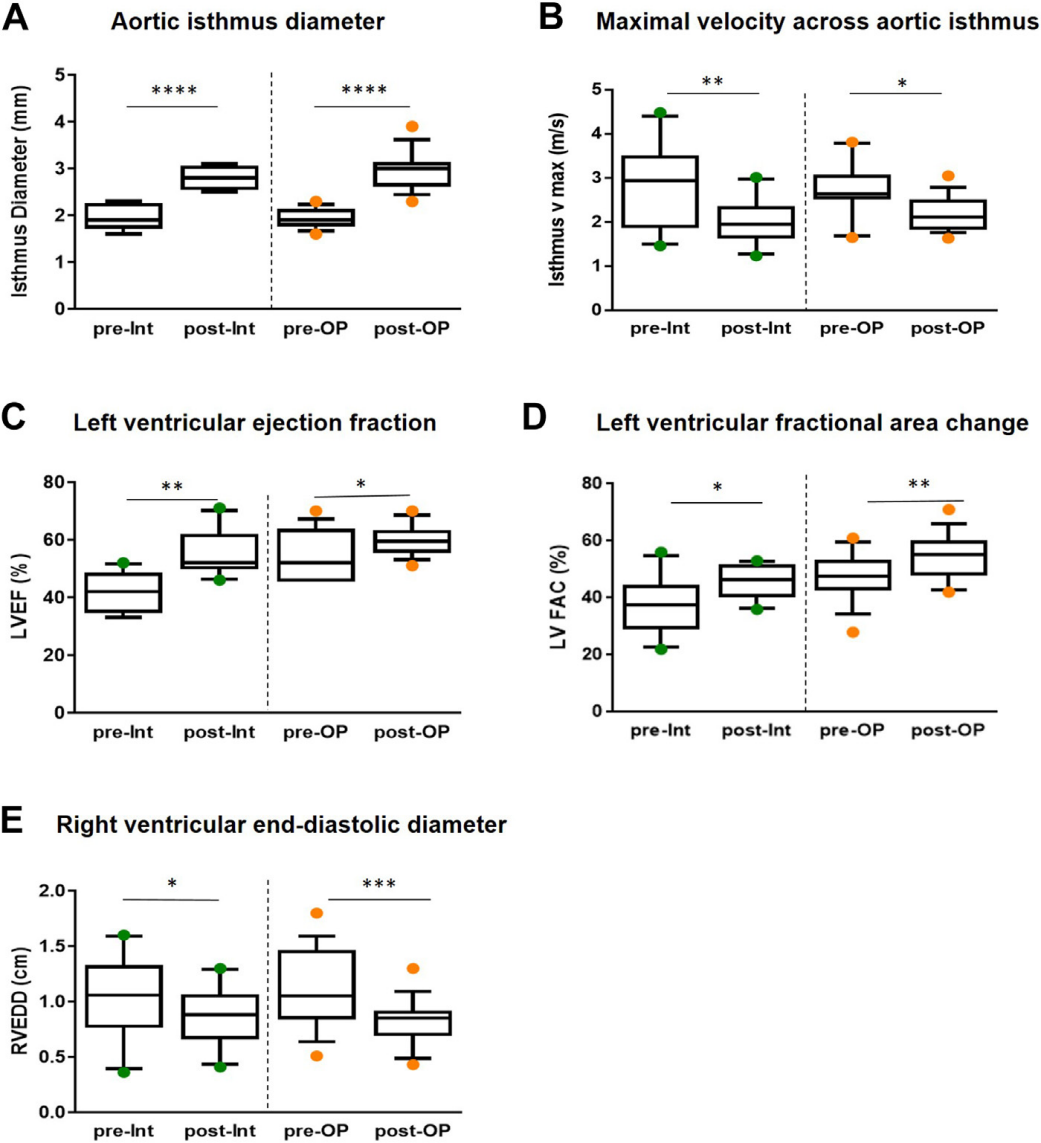
**Improvement of Echocardiographic Variables Post-CoA Treatment** All patients (n = 26) received advanced echocardiographic examinations pre- and post-therapy, at hospital admission and prior to hospital discharge, respectively. The plots show echocardiographic changes of all, the aortic isthmus (diameter, velocity) **(A and B)**, systolic LV function, represented by FAC and LVEF, Simpson, monoplane **(C and D)** as well as RV end-diastolic diameters **(E)**. The box and whisker plots show the median, interquartile range and 10th and 90th percentile. The **interrupted line between the graphs** indicates that we did not perform statistical comparisons between the cohorts. ∗*P* ≤ 0.05, ∗∗*P* ≤ 0.01, ∗∗∗*P* ≤ 0.001, ∗∗∗∗*P* ≤ 0.0001. CoA = coarctation of the aorta; FAC = fractional area change; LVEF = left ventricular ejection fraction; post-Int = post Intervention; pre-Int = pre-Intervention; post-OP = post-operation; pre-OP = pre-operation; RVEDD = right ventricular end-diastolic diameter; v max = maximal velocity.

The effectiveness of therapy was evaluated by assessing diameter and flow changes at the aortic isthmus (CoA site). Morphological outcomes similarly improved in both cohorts (catheter intervention, surgery), amounting to an increase in mean isthmus diameter from 1.9 ± 0.08 mm pre-intervention to 2.8 ± 0.07 mm post-intervention and from 1.9 ± 0.05 mm preoperatively to 3.0 ± 0.1 mm postoperatively (*P* < 0.0001) (Figure 3A, Supplemental Table 2). Peak velocity across the aortic isthmus in CW Doppler decreased post-therapy to the normal range both in the interventional cohort (from 2.8 ± 0.3 m/s to 2.0 ± 0.2 m/s; *P* = 0.009) and in the surgical cohort (from 2.7 ± 0.2 m/s to 2.2 ± 0.1 m/s; *P* = 0.016) (Figure 3B, Supplemental Table 2).

### Systolic LV and RV function recover prior to hospital discharge in neonatal CoA regardless of therapy modality (catheter intervention or surgery)

Recovery of biventricular systolic function was found in all 26 neonates with CoA after relief of LV pressure overload, as assessed by advanced echocardiography and speckle tracking analysis. Improvements were seen in both LV fractional area change in the PSAX view and LVEF using Simpson’s method in monoplane A4C view (LVEF: from 41.7% ± 2.2% to 54.9% ± 2.4% in interventional patients and from 54.4% ± 2.1% to 59.6% ± 1.4% in the surgical group) (Figures 3C and 3D). AoV-VTI increased significantly in the interventional cohort (*P* = 0.04), greatly varying in the surgical group preoperatively and postoperatively (*P* = 0.0419) (Supplemental Figure 5C). Of note, such peak velocities must be carefully interpreted when systolic LV function is severely impaired in critical CoA. The interventional patients had poorer systolic LV function at baseline (ie, LVEF <50% in 90% of the interventional patients vs 38% of the surgical patients), but even patients with LVEF <50% reached normal LVEF post-intervention. Differences in AoV-VTI between cohorts at baseline might be due to the poorer systolic LV function on admission in interventional patients, left-to-right shunt across the PDA in some of the surgical patients, and flow across the patent foramen ovale/atrial septal defects.

The RV anterior wall diameter at end diastole (RWAWd, B-Mode, PSAX) as a surrogate measure of RV mass and hypertrophy did not significantly change post-therapy (Supplemental Table 2). The RVEDD (B-Mode, PSAX), however, notably decreased in both patient cohorts (Figure 3E). Echocardiographic change of variables in individual patients during hospital stay is shown in Supplemental Figure 6.

### Improvement of myocardial contractility by means of systolic strain analysis in neonatal CoA after therapy

**Figure 4:**
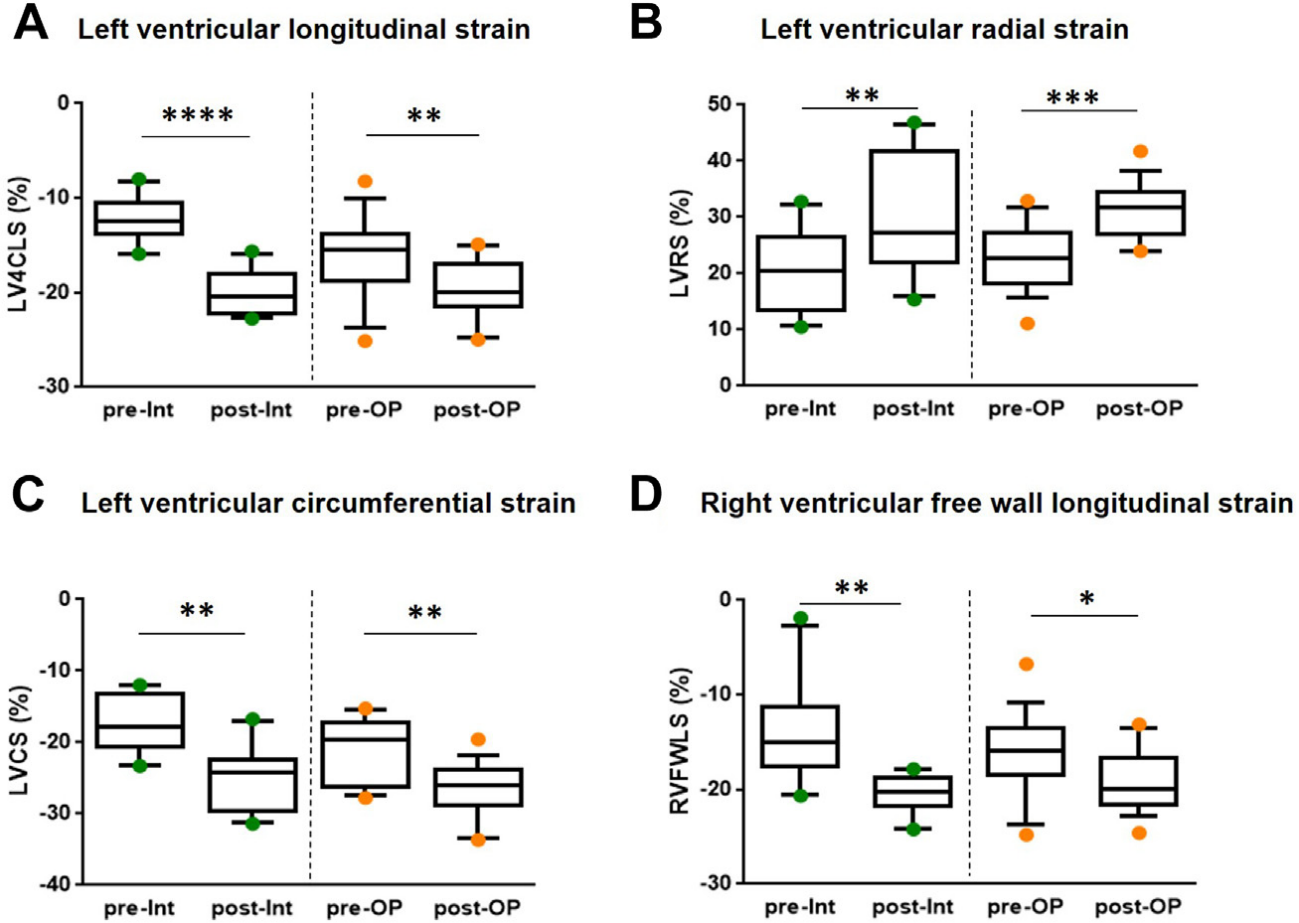
**Speckle Tracking Echocardiography and LV and RV Strain Analysis** In order to detect earlier and more modest impairment of myocardial contractility, we performed 2D speckle tracking analysis. We report recovery of biventricular function in both cohorts as observed in LV and RV myocardial strain variables **(A-D)**. The box and whisker plots show the median, interquartile range, and 10th and 90th percentile. The interrupted line between the graphs indicates that we did not perform statistical comparisons between the cohorts. ∗*P* ≤ 0.05, ∗∗*P* ≤ 0.01, ∗∗∗*P* ≤ 0.001, ∗∗∗∗*P* ≤ 0.0001. LV4CLS = LV four chamber longitudinal strain; LVCS = LV circumferential strain; LVRS = LV radial strain; post-Int = post-Intervention; pre-Int = pre-Intervention; post-OP = post-operation; pre-OP = pre-operation; RV = right ventricle; RVFWLS = RV free wall longitudinal strain.

The results of 2D-speckle tracking echocardiography and subsequent strain analysis are summarized in Supplemental Table 2. LV longitudinal strain (LV 4CLS) was −12.3% ± 0.7% for the interventional cohort and −16.3% ± 1.1% for the surgical cohort pre-therapy, rapidly normalizing post-therapy to −19.9% ± 0.8% in both groups (Supplemental Table 2, Figure 4A). Similarly, LV radial strain and the LV circumferential strain improved post-therapy, most notably in interventional patients despite larger data variance (*P* = 0.003 and *P* = 0.002, respectively) (Figures 4B and 4C).

The RV free wall longitudinal strain was also greatly decreased in most CoA patients, reaching mean values of −13.8% ± 1.7% pre-intervention and −16.1% ± 1.0% pre-surgery, considerably improving to mean values of −20.5% ± 0.6% post-interventionally and −19.1% ± 0.8% postoperatively (Supplemental Table 2, Figure 4D). The Bland-Altman plots for all strain variables in Supplemental Figure 3 illustrate good intraobserver agreement, supported by the ICCs in the figure legend.

### Improvement in LV diastolic strain variables post-therapy for neonatal CoA

**Figure 5:**
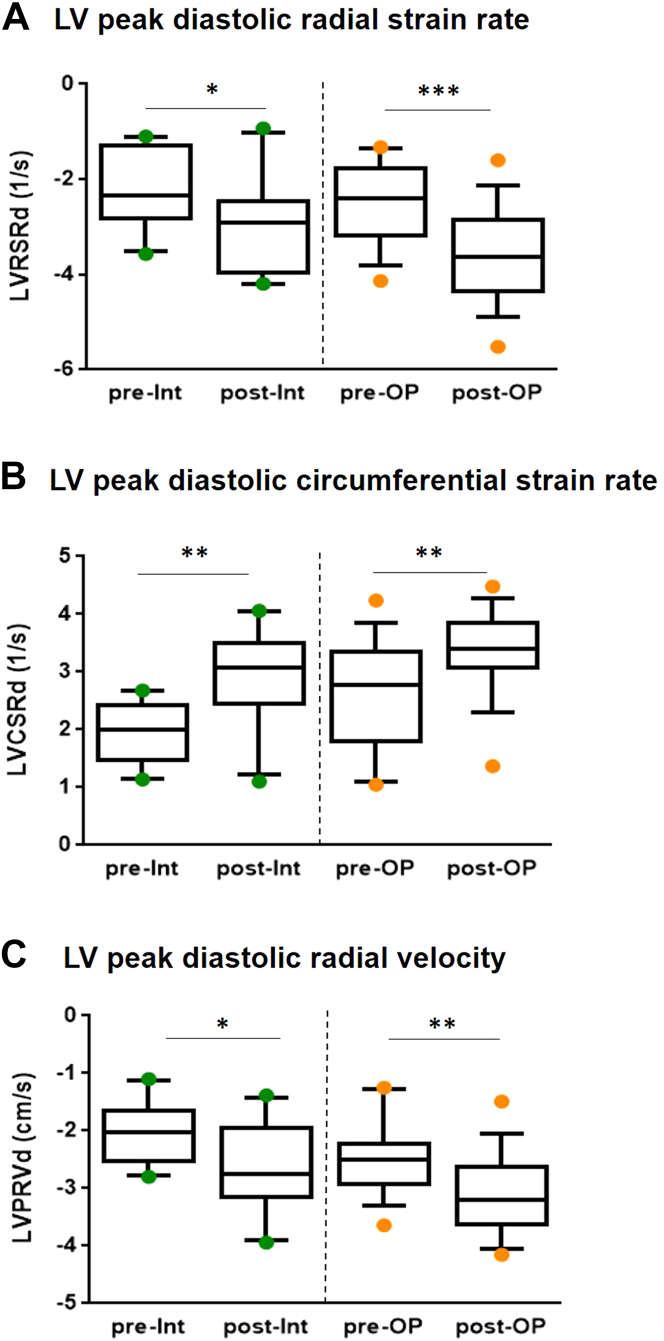
**Improvement of LV Diastolic Strain Variables Post-CoA Therapy** Patients in both cohorts reached similar diastolic strain values post CoA treatment. The plots illustrate changes in peak diastolic radial **(A)** and circumferential strain rates **(B)** as well as improvement in LV peak diastolic radial velocity **(C)**. The box and whisker plots show the median, interquartile range, and 10th and 90th percentile. The interrupted line between the graphs indicates that we did not perform statistical comparisons between the cohorts. ∗*P* ≤ 0.05, ∗∗*P* ≤ 0.01, ∗∗∗*P* ≤ 0.001. LV = left ventricle; LVCSr = LV circumferential strain rate; LVPRD = LV peak radial displacement; LVPRV = LV peak radial velocity; LVRSr = LV radial strain rate; post-Int = post Intervention; pre-Int = pre Intervention; post-OP = post-operation; pre-OP = pre-operation.

Figure 5 illustrates the change of early diastolic LV strain variables with therapy. LV peak diastolic radial strain rate improved from −2.22 ± 0.26 to −2.97 ± 0.33 in the interventional cohort and from −2.53 ± 0.21 to −3.56 ± 0.25 in the surgical group (Supplemental Table 2, Figure 5A). The LV peak diastolic circumferential strain rate also improved post-therapy, with mean Z-scores decreasing from −4.29 to −3.33 and from −3.61 to −2.93 in the interventional and surgical groups, respectively (Figure 5B). Finally, LV peak diastolic radial velocity also ameliorated in both cohorts, reaching mean values of −2.67 ± 0.25 and −3.1 ± 0.17 post-interventional and surgical treatment, respectively (Supplemental Table 2, Figure 5C). The change of strain variables in individual patients during hospital stay is shown in Supplemental Figures 7 and 8.

### Procedural adverse events

Adverse events in the interventional cohort were limited to moderate local vascular complications. Two of the interventional patients developed femoral artery thrombosis and were managed conservatively (intravenous heparin), resulting in normal leg perfusion. Another patient presented with an echocardiographically, minimally dislodged but otherwise not altered-stent. There were no direct therapeutic complications in the surgical cohort. All surgical patients, however, required placement of central venous catheters and 2 of them had central venous catheter-related sepsis requiring antibiotic treatment. There were no other major complications and no early or late deaths in either cohort.

### Clinical course and midterm outcomes of the interventional patients

Detailed information on the timeline of definitive surgical repair of the CoA in interventional patients can be found in Supplemental Figure 1 and Supplemental Table 3. The 2 patients receiving only balloon angioplasty did not present with heart failure on hospital admission and were evaluated to be stable enough for surgery soon after balloon angioplasty. One of the latter 2 infants developed a considerable pressure gradient across the CoA (26 mm Hg) and a subsequent decrease in systolic LV performance shortly after the initial intervention. Thus, the balloon angioplasty-to-surgery interval was short (approximately 1 week) in these 2 patients. The other 8 interventional patients underwent surgery at different time points after initial intervention (range: 2-24 months, n = 6) or had no surgery (42-66 months follow-up, n = 2).

### Clinical follow-up 1-year post-intervention

One year post-intervention (11 ± 2 months) all patients were clinically stable, had strong femoral pulses, and NIBPGs (10.7 ± 6 mm Hg). They demonstrated normal systolic biventricular function; only one patient had reduced diastolic LV performance in Tissue Doppler Imaging (TDI). One-half (n = 4) of the patients who were initially stented had undergone elective surgical CoA repair at our midterm follow-up 1 year post-intervention. Two of the remaining 4 patients had v max >2 m/s across the aortic isthmus, but were otherwise asymptomatic; they later underwent sequential balloon re-dilation of the stent.

## Discussion

In this study, neonates with isolated critical CoA treated with balloon dilation ± stent placement or surgery had full recovery of biventricular systolic function regardless of treatment modality or clinical status at hospital admission. Conventional echocardiographic variables such as isthmus diameter and V max across the CoA on CW Doppler improved. LV systolic function, including strain variables, normalized after relief of LV pressure overload. RV free wall longitudinal strain also normalized (Central Illustration). At 1 year after the intervention all patients were clinically stable.

**Central Illustration:**
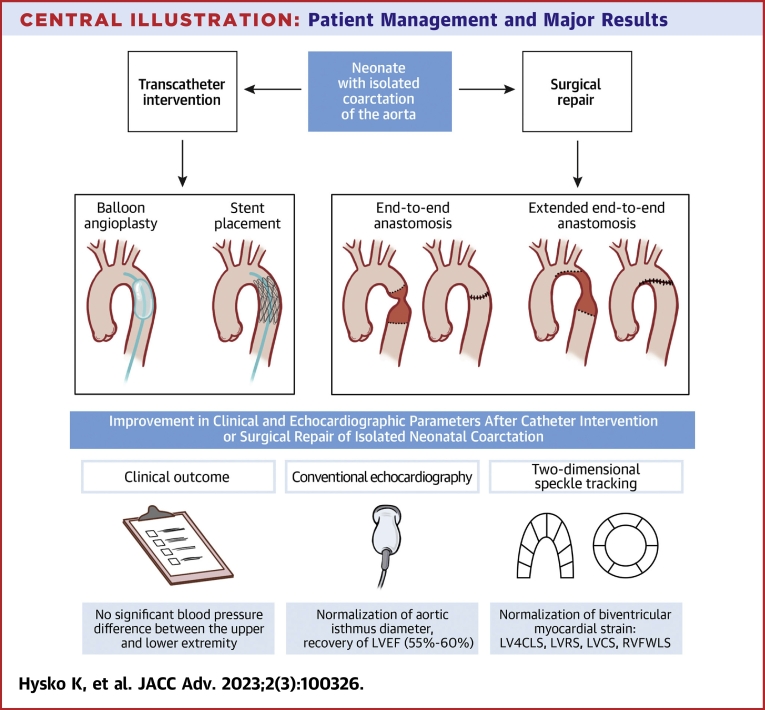
**Patient Management and Major Results** LV4CLS = LV four chamber longitudinal strain; LVCS = LV circumferential strain; LVEF = left ventricular ejection fraction; LVRS = LV radial strain; RVFWLS = RV free wall longitudinal strain.

Previous studies have described persistently impaired LV function after repair of CoA in neonates^7^^,^^8^ and non-neonatal patients.^9^, ^10^, ^11^, ^12^, ^13^ In this study, biventricular recovery of the systolic function occurred in both interventional and surgical CoA patients. All conventional echocardiographic variables related to systolic LV function significantly improved in the interventional cohort, and normalized in most patients prior to hospital discharge. Importantly, not all interventional patients improved to the same degree. For instance, the 2 patients that underwent balloon angioplasty had less improvement in LVEF post-intervention, possibly due to residual coarctation causing persistently increased pressure afterload or lower LV systolic function at baseline.

A previous study examined the dynamics of ventricular performance in healthy neonates during the first 6 to 7 weeks of life, yielding strain reference values.^14^ We comparatively assessed change of LV systolic strain variables and observed their normalization post-therapy. We also found decreased myocardial contractility of the RV in both CoA cohorts at baseline. Our speckle tracking analysis suggests that the qualitative global systolic RV dysfunction at hospital admission was not only a result of impaired interventricular septal movement but also due to decreased RV free wall contractility. Systolic RV function then improved prior to hospital discharge, possibly due to decreased LV and left atrial pressure, more favorable interventricular interaction and normalized pulmonary artery pressure (RV afterload).^4^ The discrepancy between our results and aforementioned studies^8^, ^9^, ^10^, ^11^, ^12^, ^13^, ^14^ suggests that early intervention (transcatheter or surgery) and the absence of other congenital heart defects might improve outcomes in neonatal CoA.

TDI is typically used to assess diastolic ventricular function; however, it greatly varies with age and is highly preload dependent. Previous studies^14^^,^^15^ found changes in TDI variables in the neonatal period and one report suggested that these alterations might correlate with myocardial growth and maturation and changing hemodynamics of the LV.^16^ We do not routinely perform TDI examinations on neonates in our center because of the aforementioned caveats, so in this study, we primarily focused on novel strain variables to assess LV diastolic performance. We found improvement of diastolic LV function post-therapy by means of changes in LV peak diastolic radial and circumferential strain rate as well as LV peak radial velocity, patients in both cohorts reached similar strain values post-therapy (Figure 5, Supplemental Table 2). Of the 3 LV diastolic strain variables we focused on, only the Boston Children’s Hospital z-scores of the LV peak diastolic circumferential strain rate are available to date. The latter variable considerably improved but did not completely normalize in either cohort post-therapy, indicating some degree of persistent diastolic LV dysfunction pre-hospital discharge, similarly to previous publications, in which diastolic performance was evaluated by TDI.^8^, ^9^, ^10^^,^^14^^,^^15^ Furthermore, the large variance of normal range data in pediatric patients for strain variables^17^^,^^18^ prevents us from making a final statement on the magnitude and clinical significance of the improvement of the diastolic LV function immediately after CoA treatment.

We found a trend toward decreasing biventricular wall thicknesses with pressure unloading, and a statistically significant decrease in RVEDD as an indicator of RV dilation and its later return to normal range values (Figure 3F). Larger cohorts are needed to properly identify more subtle changes in ventricular dimensions. Nevertheless, we feel our data emphasize biventricular distress caused by CoA and the importance of close echocardiographic follow-ups after hospital discharge.

The midterm outcomes of the interventional cohort were very promising: at 1 year post-intervention, 6 of the 10 patients had undergone elective surgical repair in excellent condition and the remaining 4 were asymptomatic even 12 to 18 months after catheter intervention. Of the latter, 2 have not had surgery as of December 2022. None of the patients presented with heart failure at any of our follow-up appointments after the initial interventional treatment, thus emphasizing the role of initial percutaneous therapy as an excellent mode of clinical stabilization. One of the most decisive factors regarding the intervention-to-surgery period is the presence of concomitant cardiovascular lesions, which in our CoA study population were limited to bicuspid aortic valve in 30% and aortic arch hypoplasia in 40% of the interventional patients. Two of the 4 interventional patients with aortic arch hypoplasia had borderline transverse arch diameters at hospital admission; prior intervention was considered less concerning for a possible future aortic arch correction than a preceding surgical repair of the CoA (Supplemental Figure 1). Moreover, there are data suggesting growth of the aortic arch to normal dimensions after CoA repair.^19^ Only one of the 2 patients with aortic arch hypoplasia required early surgical aortic arch repair. While there is a possibility of stent advancement into the aortic arch in interventional patients, we generally opt for a 2-step treatment strategy in severely compromised neonates with critical CoA at our institution, first interventionally addressing the CoA as the most relevant lesion, followed by the final surgical repair of the aortic arch hypoplasia and CoA after patient stabilization and recovery.

The coronary stents that were implanted in the neonatal period will eventually elicit an aortic isthmus stenosis in the growing aorta and associated phenomena such as increased NIBPGs, flow acceleration across the stent, and stent fractures. Depending on stent expandability, these patients undergo percutaneous stent balloon re-dilation to match somatic growth and/or receive elective surgical therapy at our institution. Hence, balloon dilation ± stent placement should not be regarded as destination therapy but rather as a mode of clinical stabilization and a bridge to definitive surgical CoA repair. Newer developments such as breakable stents may have the potential to become an alternative and long-term treatment modality in selected neonates with isolated CoA.^20^, ^21^, ^22^ Once approved for use in neonates, these breakable stents may allow for lifelong transcatheter therapy/re-dilation up to adult dimensions; however, follow-up data beyond 1 year are pending.

### Study limitations

In this study, we focused on neonates with isolated CoA. However, there is a number of congenital heart defects associated with CoA. Implications of concomitant cardiac/cardiovascular lesions on (interventional) patient outcomes should be the focus of future studies. Another limitation of this study was the small cohort size, as is often the case in pediatric studies. Given our therapeutic algorithm and retrospective study design, we analyzed changes within each treatment group, but were not able to compare the efficacy of the two treatment modalities directly. Nevertheless, we feel our data illustrate similarity in treatment outcome, and the importance of emergency catheter interventions as rescue treatment in critical neonatal CoA.

## Conclusions

Both primary catheter intervention and surgical repair lead to recovery of biventricular systolic function and improvement of LV diastolic function in neonates with critical, isolated CoA, regardless of treatment modality or severity of cardiac dysfunction at hospital admission. Thus, percutaneous transcatheter intervention (balloon angioplasty ± stent) is a feasible alternative to initial surgical repair, particularly for those newborn infants who present with severely impaired systolic LV function or even decompensated heart failure/cardiac shock.

PERSPECTIVES **COMPETENCY IN PATIENT CARE AND PROCEDURAL SKILLS:** Our encouraging outcomes highlight the feasibility of transcatheter treatment of critically ill neonates with isolated CoA, including implantation of coronary stents. Nevertheless, finite stent expandability is a limitation of such a treatment approach.

**TRANSLATIONAL OUTLOOK:** Mid- and long-term outcome regarding safety, efficacy, and rates of reintervention after any stent placement for neonatal CoA beyond 1 year should be further investigated.

## Funding support and author disclosures

This study was supported by the 10.13039/501100001659German Research Foundation DFG KFO 311 “Pre-Terminal Heart and Lung Failure–Unloading and Repair” (HA4348/6-2 to G.H.). The authors have reported that they have no relationships relevant to the contents of this paper to disclose.
