# Supplementary material for: Recovery of Biventricular Function After Catheter Intervention or Surgery for Neonatal Coarctation of the Aorta
Source: JACC Adv. 2023 May 10;2(3):100326. doi: 10.1016/j.jacadv.2023.100326 (PMC11198406; doi:10.1016/j.jacadv.2023.100326)
Supplement: Supplemental Figures 1-8 and Tables 1-3 [file mmc1.pdf]

SUPPLEMENTAL APPENDIX

INTRODUCTION

Coarctation of the aorta (CoA) is a common congenital obstructive anomaly of the aortic arch, defined as a juxtaductal narrowing of the aortic isthmus. It affects 4 per 10,000 births and accounts for 5-8% of all congenital heart diseases[1,2]. There is considerable variation in the severity of the coarctation and associated congenital heart defects such as atrial and ventricular septal defects or cardiovascular lesions such as aortic arch hypoplasia, bicuspid aortic valve (BAV) or patent ductus arteriosus (PDA)[3,4].

Critical CoA typically manifests in neonates after a sudden constriction or closure of the ductus arteriosus and is characterized by an imminent or actual cardiogenic shock. The severity of cardiovascular decompensation and clinical manifestation depends on the rapidity of closure of the PDA, the diameter of the isthmus and the time of occurrence postnatally. While some neonates present with tachypnea, increased respiratory effort, restlessness, irritability, excessive perspiration and feeding poorly, others deteriorate rather rapidly. They may exhibit progressive respiratory distress, tachycardia and weak to absent femoral pulses as a symptom of systemic hypoperfusion[5]. In order to re-open or vasodilate the PDA, thus securing systemic blood flow, prostaglandin (PGE1) is often administered via continuous intravenous infusion, in addition to ICU measures such as inotropic support and correction of acidosis[6].

Once patients are clinically stable, they undergo surgical repair of the CoA as the current gold standard in CoA treatment[7-9]. Transcatheter balloon dilation of the CoA ± stent placement are not routinely performed in neonates because of relatively high rates of restenosis and local complications such as femoral arterial injury and aneurysms[10-12]. There are, however, few reports on favorable outcomes in neonates with isolated coarctation presenting with cardiogenic shock/ severe left ventricular dysfunction who underwent emergency surgical therapy[13]. Other studies suggest that surgery unequivocally yields better intermediate and midterm results in neonates[8,9].

Supplemental Table 1. Individual patient and procedure characteristics in the interventional cohort.

ID	Age (days)	Sex (M/F)	Height (cm)	Weight (kg)	BSA (m^2)	Serum Lactate (mmol/l)	Arterial Access	Sheath	Ballon Diameter (mm)	Stent	Stent Diameter (mm)	Stent Length (mm)
1	3	M	55.00	3.50	0.22	2.00	RFA	Terumo 4F, 7 cm	4mm NcEmerge max. 8atm; 5mm Saber max. 9atm			
2	3	M	51.00	2.60	0.19	10.90	RFA	Terumo 4F, 7 cm		Integrity coronary stent system. Medtronic Vascular	4	12
3	7	M	51.00	3.10	0.20	15.00	LSA	PediaVascular 3.3F, 7cm	4mm Nc Emerge max. 12atm	Integrity coronary stent system. Medtronic Vascular	4.5	12
4	8	M	47.00	2.30	0.17	5.40	RFA	Terumo 4F, 7 cm		Integrity coronary stent system. Medtronic Vascular	4	9
5	9	M	50.00	3.60	0.21	1.50	RFA	Terumo 4F, 7 cm	4 mm Savvy max. 10atm	Driver Sprint. Medtronic Vascular	4.5	12
6	18	F	46.00	2.20	0.16	2.90	LFA	Terumo 4F, 7 cm		Integrity coronary stent system. Medtronic Vascular	4	12
7	21	F	50.00	3.30	0.20	6.30	RFA	Terumo 4F, 7 cm		Integrity coronary stent system. Medtronic Vascular	4	9
8	22	M	50.00	3.70	0.21	10.20	RFA	Terumo 4F, 7 cm		Integrity coronary stent system. Medtronic Vascular	3.5	12
9	27	M	52.00	4.00	0.23	1.60	RFA	Cordis Avanti 5 F		Formula 414. Cook Peripheral Stent	5	12
10	28	M	53.00	4.30	0.24	2.00	RFA	Terumo 4F, 7 cm	6 mm Tyshak II max. 4 atm			

Patients 1 and 10 only received balloon dilation. In patients 3 and 5 additional stent placement was necessary due to unsatisfactoryoutcomes after initial balloon dilation. Owing to a lack of readily available commercial intravascular stents approved for use in pediatric patients, balloon- expandable, premounted coronary stent systems are typically used in our center. Abbreviations: LFA, left femoral artery; LSA, left subclavian artery; RFA,right femoral artery.

Supplemental Table 2. Advanced echocardiography and strain analysis.

	Interventional Cohort (n= 10)			Surgical Cohort (n=16)		
	Pre-Int	Post-Int	p value	Pre-OP	Post-OP	p value
**ECHOCARDIOGRAPHY**						
**Aorta**						
Aortic Isthmus Diameter (mm)	1.9 ± 0.274 (0.08)	2.8 ± 0.226 (0.07)	<0.0001	1.9 ± 0.192 (0.05)	3 ± 0.396 (0.1)	<0.0001
Aortic Isthmus Ratio (Isthmus/DAO)	0.4 ± 0.06 (0.02)	0.6 ± 0.075 (0.02)	<0.0001	0.3 ± 0.046 (0.01)	0.55 ± 0.082 (0.02)	<0.0001
Isthmus v max (m/s)	2.8 ± 1.05 (0.3)	2.0 ± 0.52 (0.2)	0.008	2.7 ± 0.62 (0.2)	2.2 ± 0.38 (0.1)	0.016
AoV VTI (cm)	12.9 ± 4.40 (1.4)	16.3 ± 3.065 (1.0)	0.041	16.6± 8.104 (2.0)	18.9 ± 8.033 (2.0)	0.0419
**Left ventricular function**						
LVEF, Simpson monoplane (%)	41.7± 6.95 (2.2)	54.9 ± 7.69 (2.4)	0.003	54.4 ± 8.29 (2.1)	59.6 ± 5.451 (1.4)	0.045
LVEF, area/length (%)	44.2 ± 9.2 (2.9)	54.7 ± 5.46 (1.7)	0.011	57.7 ± 6.69 (1.7)	61.6 ± 6.55 (1.6)	ns(0.078)
LV FAC (%)	37.6 ± 5.316 (3.0)	45.9 ± 5.782 (1.8)	0.02	47.4 ± 8.13 (2.0)	54.9 ± 7.702 (1.9)	0.007
**Left ventricular size**						
LVEDD, PSAX (cm)	1.9 ± 0.362 (0.12)	1.9 ± 0.45 (0.14)	ns(0.419)	1.72 ± 0.29 (0.07)	1.92 ± 0.179 (0.04)	0.003
FS, PSAX (%)	27.2 ± 6.515 (2.17)	29.1 ± 8.19 (3.5)	ns(0.808)	32.1 ± 8.85 (2.21)	33.5 ± 7.127 (1.78)	ns(0.618)
IVSD/LVPWD	1.1 ± 0.217 (0.07)	1.17 ± 0.233 (0.07)	ns(0.227)	1.02 ± 0.21 (0.05)	1.16 ± 0.244 (0.06)	ns(0.082)
RWT*	0.33 ± 0.084 (0.03)	0.37 ± 0.115 (0.04)	ns(0.221)	0.42 ± 0.11 (0.03)	0.37 ± 0.06 (0.01)	ns(0.136)
**Right ventricular size**						
RVAWD, PSAX (cm)	0.33 ± 0.09 (0.03)	0.29 ± 0.06 (0.02)	ns(0.09)	0.30 ± 0.05 (0.01)	0.30 ± 0.05 (0.01)	ns(0.678)
RVEDD, PSAX (cm)	1.03 ± 0.38 (0.13)	0.88 ± 0.27 (0.08)	0.048	1.1 ± 0.35 (0.08)	0.81 ± 0.202 (0.05)	0.001
RV/LV end-systolic ratio	0.68 ± 0.38 (0.12)	0.62 ± 0.31 (0.1)	ns(0.734)	0.99 ± 0.55 (0.14)	0.55 ± 0.201 (0.05)	0.002
**STRAIN ANALYSIS**						
**Left ventricular strain**						
LV4CLS (%)	-12.3 ± 2.32 (0.7)	-19.9 ± 2.39 (0.8)	<0.0001	-16.3 ± 4.41 (1.1)	-19.9 ± 3.09 (0.8)	0.003
LVRS (%)	20.4 ± 7.24 (2.3)	29.9 ± 10.4 (3.3)	0.003	22.8 ± 5.68 (1.4)	31.3 ± 5.09 (5.1)	0.0002
LVRSr (1/s)	1.72 ± 0.65 (0.21)	2.23 ± 0.82 (0.26)	0.03	1.88 ± 0.62 (0.15)	2.45 ± 0.43 (0.11)	0.002
LVPRD (mm)	2.06 ± 0.55 (0.18)	2.53 ± 0.46 (0.14)	0.015	2.23 ± 0.50 (0.12)	2.75 ± 0.51 (0.13)	0.006
LVPRV (cm/s)	1.54 ± 0.44 (0.12)	1.97 ± 0.69 (0.18)	0.037	1.81 ± 0.51 (0.13)	2.24 ± 0.28 (0.06)	0.004

	Interventional Cohort (n= 10)			Surgical Cohort (n=16)		
	**Pre-Int**	**Post-Int**	**p value**	**Pre-OP**	**Post-OP**	**p value**
**STRAIN ANALYSIS**						
**Left ventricular strain**						
LVCS (%)	-17.4 ± 3.99 (1.3)	-24.8 ± 4.68 (1.5)	0.002	-18.0 ± 4.48 (1.1)	-26.3 ± 3.76 (0.9)	0.006
LVCSr (1/s)	-1.53 ± 0.41 (0.13)	-2.25 ± 0.83 (0.26)	0.012	-1.83 ± 0.57 (0.14)	-3.27 ± 0.55 (0.14)	<0.0001
**Right ventricular strain**						
RVFWLS (%)	-13.8 ± 3.49 (1.7)	-20.5 ± 1.99 (0.6)	0.002	-16.1 ± 4.29 (1.0)	-19.1 ± 3.19 (0.8)	0.03

All patients (n=26) received advanced echocardiographic examination pre- and post- therapy, at hospital admission and shortly before hospital discharge respectively, with echo-to-echo intervals of 10±6 days for the interventional patients and 11±4 days for the surgical patients. *Relative wall thickness was determined as the twofold of the left ventricular posterior wall thickness divided by left ventricular end- diastolic diameter. All measurements are presented as mean ± SD and SEM in parenthesis below. A t-test and Wilcoxon's signed-rank test were applied for parametric and non-parametric data respectively. Abbreviations: AoV VTI, aortic valve velocity time integral; FAC, fractional area change; FS, fractional shortening; IVSD/LVPWD, interventricular septal thickness-to- left ventricular posterior wall thickness ratio; LV, left ventricle; LVCS, LV circumferential strain; LVCSr, LV circumferential strain rate; LVEDD, LV end-diastolic diameter; LVEF, LV ejection fraction; LVPRD, LV peak radial displacement; LVPRV, LV peak radial velocity; LVRS, LV radial strain; LVRSr, LV radial strain rate; LV4CLS LV 4 chamber longitudinal strain; PSAX, parasternal short axis; RV, right ventricle; RVAWD, RV anterior wall thickness at end diastole; RVEDD, RV end-diastolic diameter; RVFWLS, RV free wall longitudinal strain; RWT, relative wall thickness; v max, maximal velocity.

Supplemental Table 3. Clinical course and echocardiographic status in infants of the interventional cohort.

	**Post-intervention (n=10)**	**Pre-surgery (n=10)**
**DEMOGRAPHICS**		
Age (months)	0.77 ± 0.39	18.33 ± 7
Sex-Male (n)(%)	12 (75%)	12 (75%)
Height (m)	0.5 ± 0.3	0.69 ± 0.17
Weight (kg)	3.3 ± 0.7	8.0 ± 4.3
BSA (m^2)	0.21 ± 0.025	0.38 ± 0.153
NIBPGs (mm Hg)	9.2 ± 10.2	16.5 ± 9.9
**ECHOCARDIOGRAPHY**		
**Aorta**		
Isthmus v max (m/s)	2.0 ± 0.05 (0.17)	2.78 ± 0.77 (0.25)
Isthmus PG max (mm Hg)	17.36 ± 9.05 (2.9)	31.7 ± 9.05 (5.8)
**Left ventricular systolic function**		
LV FAC (%)	45.9 ± 5.78 (1.83)	53.3 ± 9.76 (3.1)
LVEF, Simpson, biplane	55.2 ± 12.21 (3.1)	65 ± 10.04 (3.35)
**STRAIN ANALYSIS**		
**Left ventricular strain**		
LV4CLS (%)	-19.9 ± 2.39 (0.8)	-20.73 ± 3.4 (1.1)
LVRS (%)	29.9 ± 10.4 (3.3)	31.0 ± 11.62 (3.7)
LVCS (%)	-24.8 ± 4.68 (1.5)	-26.48 ± 4.37 (1.8)
**Right ventricular strain**		
RVFWLS (%)	-20.5 ± 1.99 (0.6)	-20.1 ± 5.9 (1.2)

We summarized data at hospital discharge after initial intervention and prior to definitive surgical CoA correction. Two interventional patients have not yet undergone surgery, thus we analyzed their last echocardiographic examinations performed at our institution. All measurements are presented as mean ± SD and SEM in parenthesis below. Abbreviations: BSA, body surface area; FAC, fractional area change; LV, left ventricle; LVCS, LV circumferential strain; LVEF, left ventricular ejection fraction; LVRS, LV radial strain; LV4CLS LV 4 chamber longitudinal strain; NIBPGs, non-invasive systolic blood pressure gradient; PG max, peak pressure gradient across the isthmus measured in CW Doppler; RV, right ventricle; RVFWLS, RV free wall longitudinal strain; v max, maximal velocity.

Supplemental Figure 1. Study flow chart

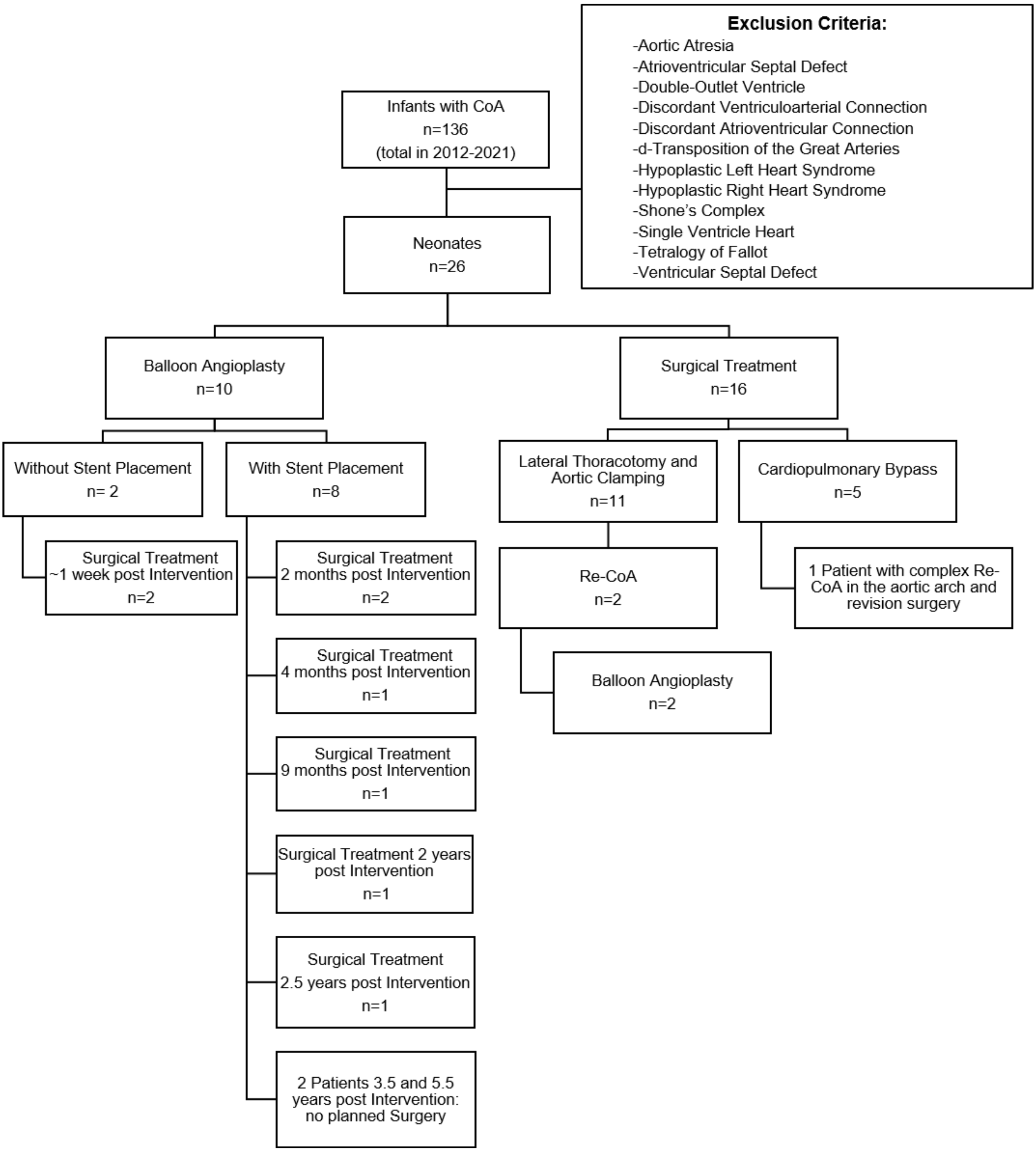

This flowchart illustrates the inclusion and exclusion criteria, the treatment our patients received and when present, events of re-coarctation of the aorta. The interventional patients received definitive surgical therapy at different times points post-intervention and two patients have not undergone surgical CoA correction yet, respectively 3.5 and 5.5 years post initial balloon angioplasty and stent placement.

Supplemental Figure 2. Representative examples of LV longitudinal strain analysis pre-and post-therapy

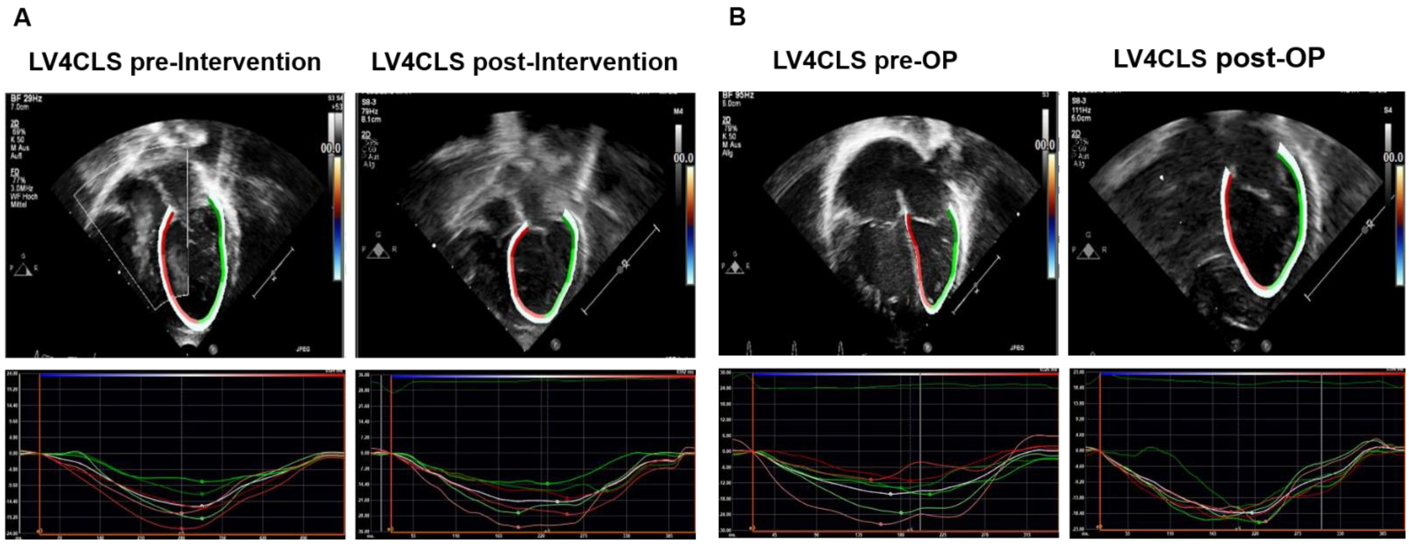

This figure shows representative echocardiographic strain images and improvement of left ventricular longitudinal strain post-intervention **(A)** and post-surgery **(B)**. The region of interest, in this case the LV, was divided into six segments and the TomTec 2D CPA (2D Cardiac Performance Analysis, TomTec Imaging systems, Unterschleissheim, Germany) extracts peak systolic values, symbolized by the dots in the strain curves.

Supplemental Figure 3. Low intraobserver variability in strain variables

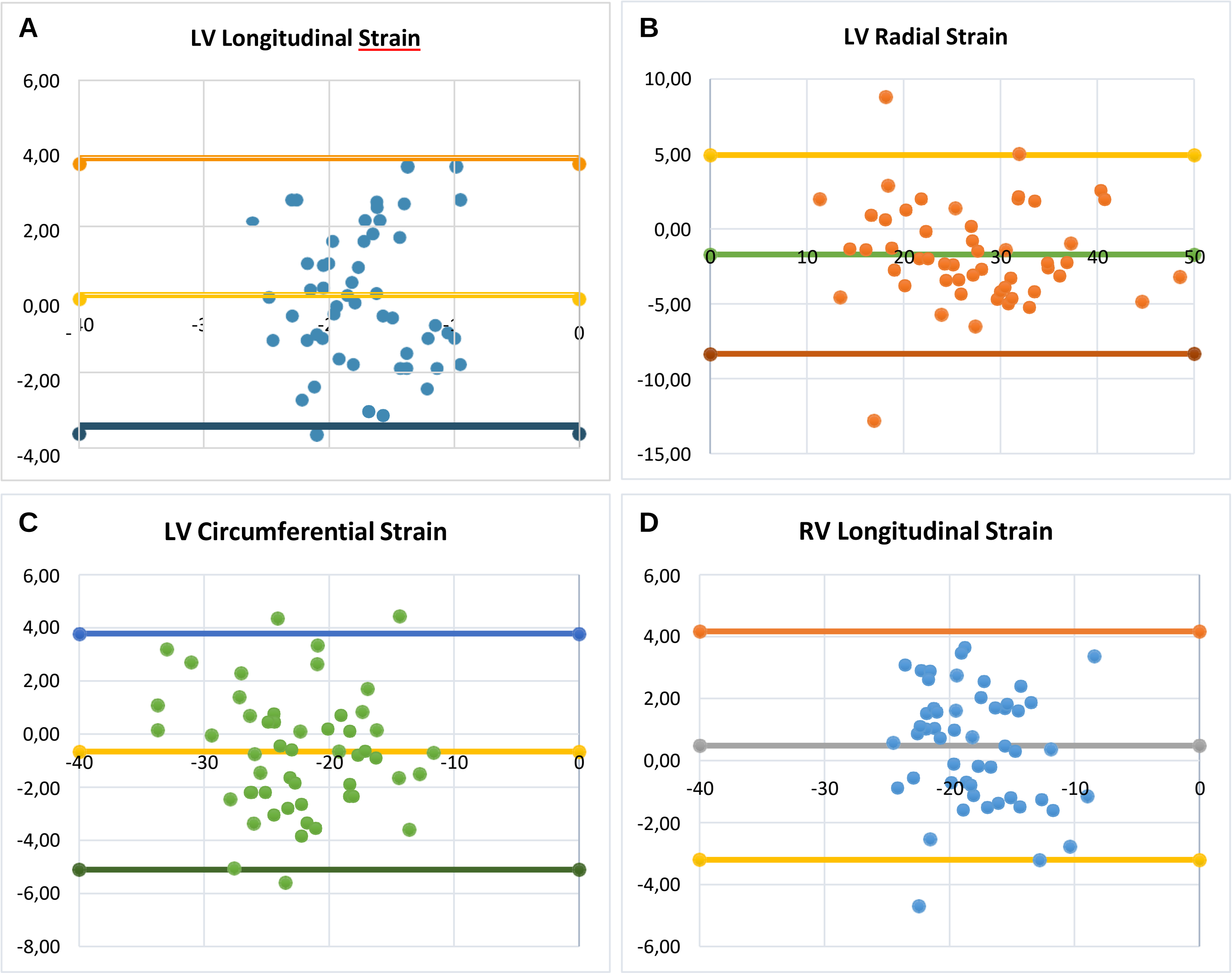

Bland-Altman plots for strain variables; the lines represent the average differences and lower and higher confidence intervals. Single dots represent strain values of individual patients (both rounds of measurements).

(A) LV Longitudinal Strain: Average Difference= 0.17, Lower CI= -3.43, Higher CI= 3.77; ICC = 0.913, 95%CI [0.854, 0.949], excellent reliability

(B) LV Radial Strain: Average Difference=-1.71 , Lower CI=-8.34, Higher CI= 4.92; ICC=0.898, 95%CI [0.786, 0.948], good reliability

(C) LV Circumferential Strain: Average Difference= -0.66, Lower CI= -5.10, Higher CI=3.78; ICC=0.906, 95%CI [0.838, 0.945], excellent reliability

(D) RV Free Wall Longitudinal Strain: Average Difference= 0,48, Lower CI= -3,20, Higher CI= 4.17 ICC=0.89, 95%CI [0.814, 0.935], good reliability

Abbreviations: CI, confidence interval; ICC, intraclass correlation coefficient; LV, left ventricle; RV, right ventricle

Supplemental Figure 4. Correlations of pressure gradients with PDA/Aortic isthmus diameter and LVEF

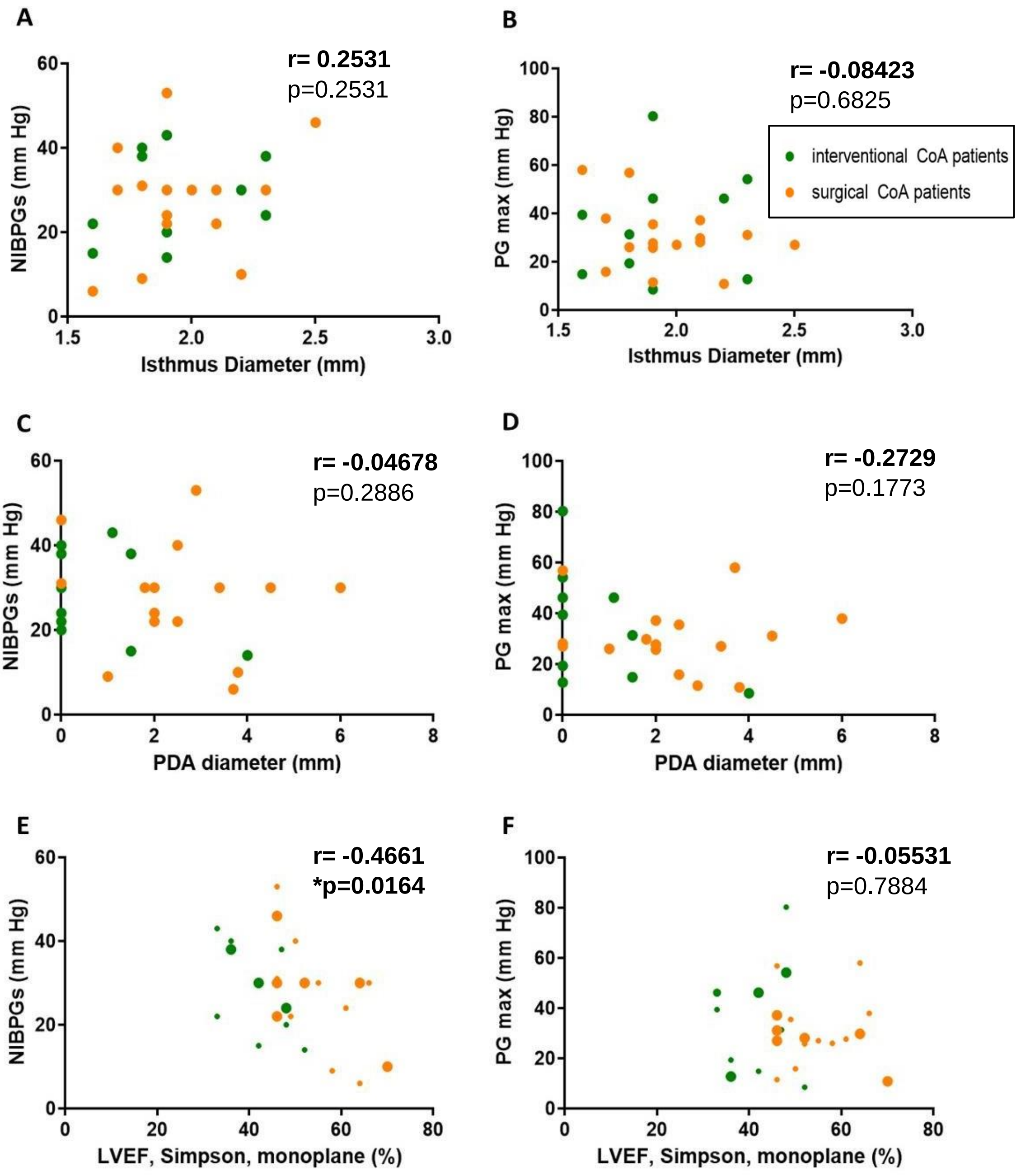

NIBPGs between the right upper and lower extremity and PG max do not correlate with isthmus and PDA size **(A-D)**. NIBPGs correlates more strongly with left ventricular performance than PG max, independently of isthmus diameter **(E,F).** Of note, some patients with poor LVEF (<50%) have lower PG max despite narrower CoA, suggesting decreasing magnitude of the pressure gradient with LV dysfunction or low cardiac output. In panels **E** and **F** dot size correlates with the isthmus diameter, smaller dots symbolizing isthmus diameter <2mm and bigger dots representing patients with isthmus diameters between 2 and 2.5mm. Green dots represent individual interventional patients and orange dots depict individual surgical patients. Abbreviations: LVEF, left ventricular ejection fraction; Non-invasive systolic blood pressure gradient between the right upper and lower extremity, systolic non-invasive blood pressure gradient; PDA, patent ductus arteriosus; PG max, maximal pressure gradient across the aortic isthmus in CW Doppler.

Supplemental Figure 5. Additional echocardiographic characterization of isthmus changes and left ventricular performance

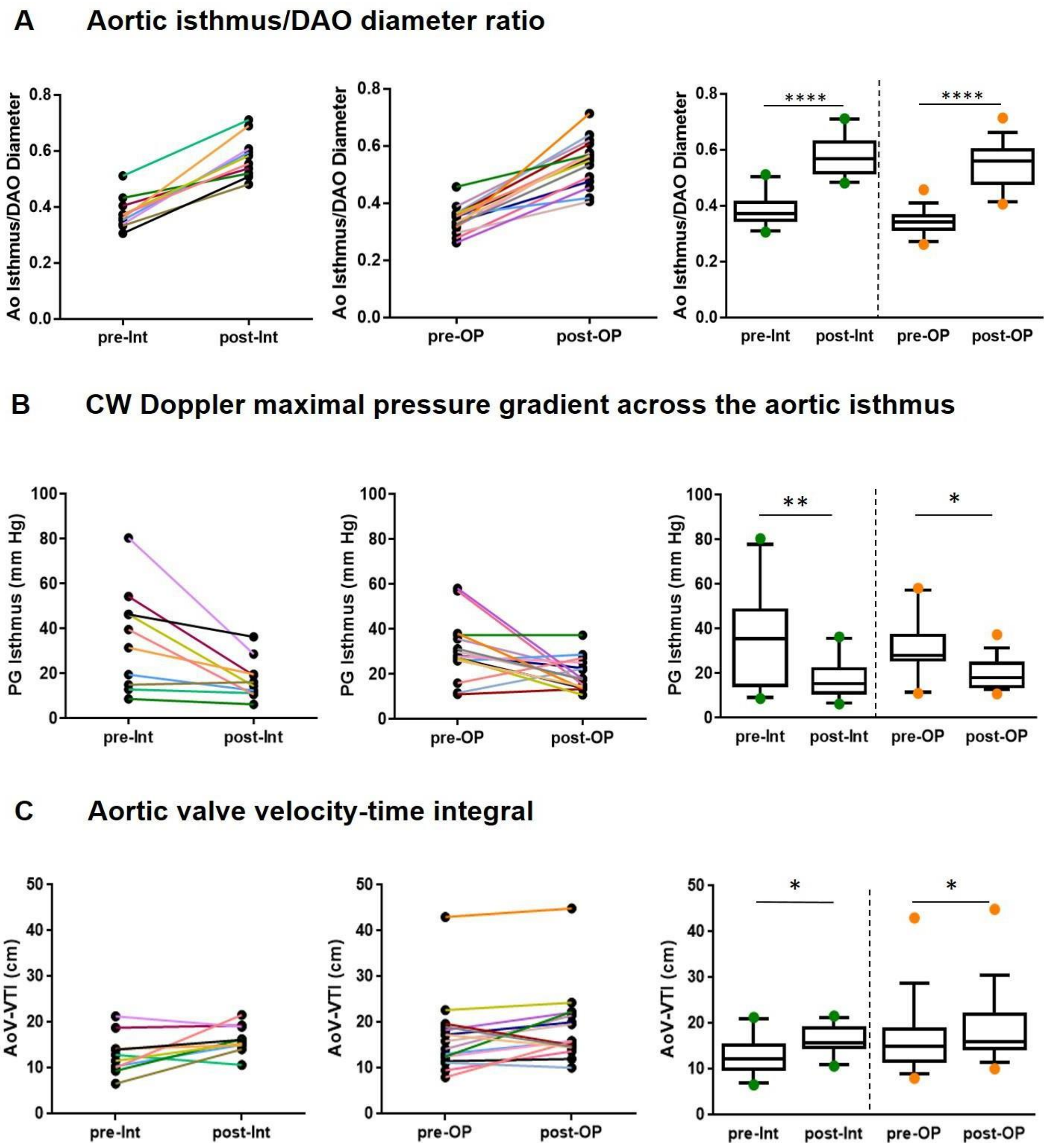

The plots show similar changes at CoA-site in both cohorts regarding isthmus dimensions and the maximal pressure gradient across the aortic isthmus **(A,B).** Change in the aortic valve- velocity time integral was statistically significant in the interventional cohort and varied greatly in surgical patients pre- and postoperatively **(C)**. Graphs in the first and second column illustrate parameter progression during hospital stay. The box and whisker plots in the third column show the median, interquartile range and 10th and 90th percentile. The scatter dot plots in the fourth column illustrate the mean with the 95% confidence interval for the mean. Green dots represent individual interventional patients and orange dots represent individual surgical patients. Student's t-test was performed on parametric data and Wilcoxon paired two-tailed t-test on non-parametric data. P-Values < 0.05 were considered significant. Abbreviations: AoV VTI, aortic valve velocity time integral; PG Isthmus, maximal pressure gradient across the aortic isthmus in CW Doppler; pre-Int, pre Intervention; post-Int, post Intervention; pre-OP, pre-operation; post-OP, post-operation.

Supplemental Figure 6. Improvement of echocardiographic variables post-CoA treatment

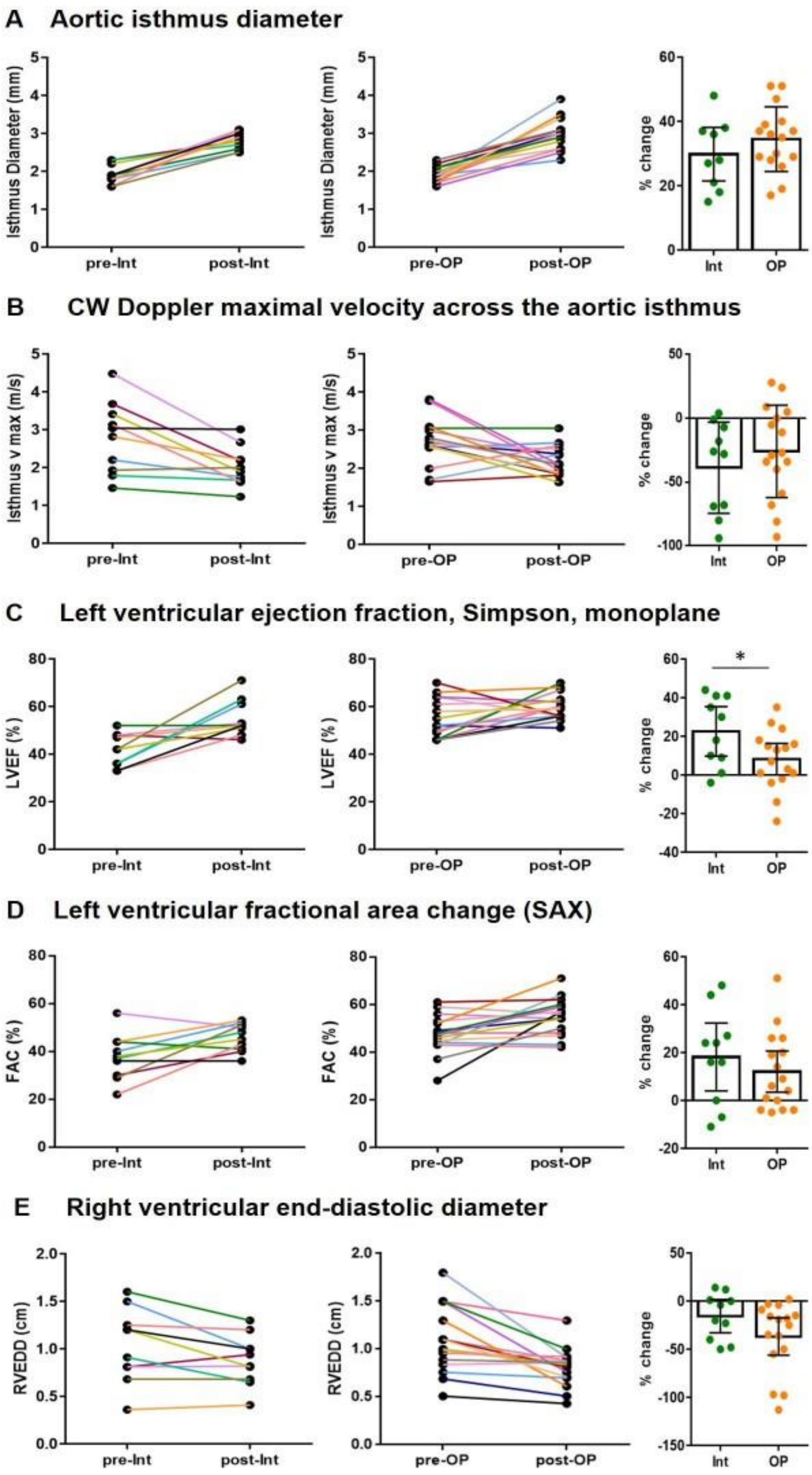

All patients (n=26) received advanced echocardiographic examinations pre- and post-therapy, at hospital admission and prior to hospital discharge, respectively. The plots show echocardiographic changes at CoA-site (**A,B**), in systolic LV function, represented by FAC and LVEF, Simpson, monoplane (**D,E**) as well as RV end-diastolic diameters (**F**). Graphs in the first and second column illustrate variable progression during hospital stay. The scatter dots plots in the third column illustrate the mean % change with the 95% confidence interval for the mean. Green and orange dots represent individual interventional and surgical patients, respectively. Abbreviations: FAC, fractional area change; LVEF, left ventricular ejection fraction; pre-Int, pre Intervention; post-Int, post Intervention; pre-OP, pre-operation; post-OP, post-operation; RVEDD, right ventricular end-diastolic diameter; v max, maximal velocity.

Supplemental Figure 7. Speckle tracking echocardiography and left and right ventricular strain analysis

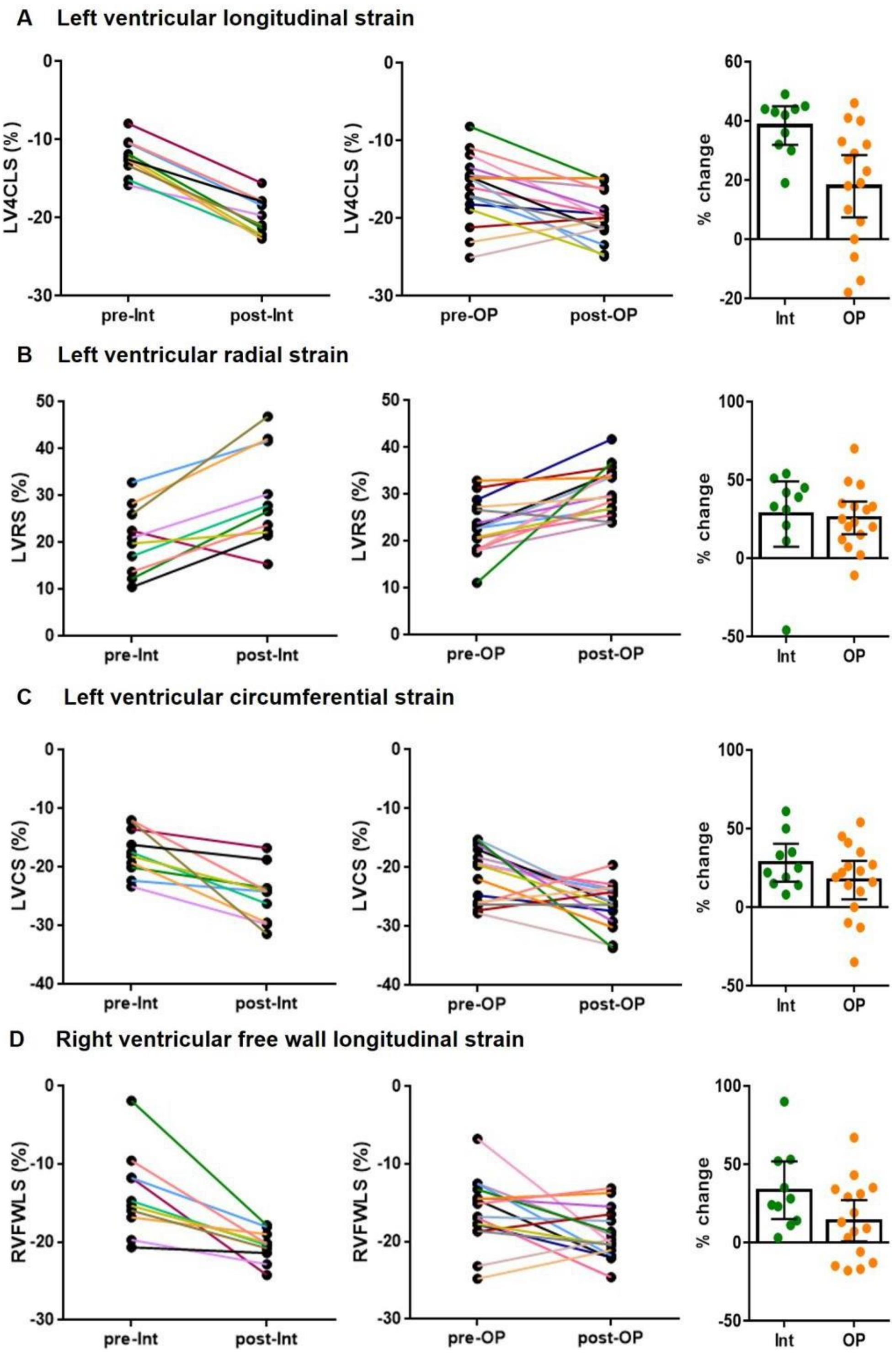

. In order to detect earlier and more modest impairment of myocardial contractility, we performed 2D speckle tracking analysis. We report recovery of biventricular function in both cohorts as observed in LV and RV myocardial strain variables (**A,B,C,D**). Graphs in the first and second column illustrate variable progression during hospital stay. The scatter dots plots in the third column illustrate the mean with the 95% confidence interval for the mean. Green and orange dots represent individual interventional and surgical patients, respectively. Abbreviations: LV, left ventricle; LVCS, LV circumferential strain; LVRS, LV radial strain; LV4CLS LV four chamber longitudinal strain; pre-Int, pre Intervention; post-Int, post Intervention; pre-OP, pre-operation; post-OP, post-operation; RV, right ventricle; RVFWLS, RV free wall longitudinal strain.

Supplemental Figure 8. Improvement of LV diastolic strain variables post-CoA therapy

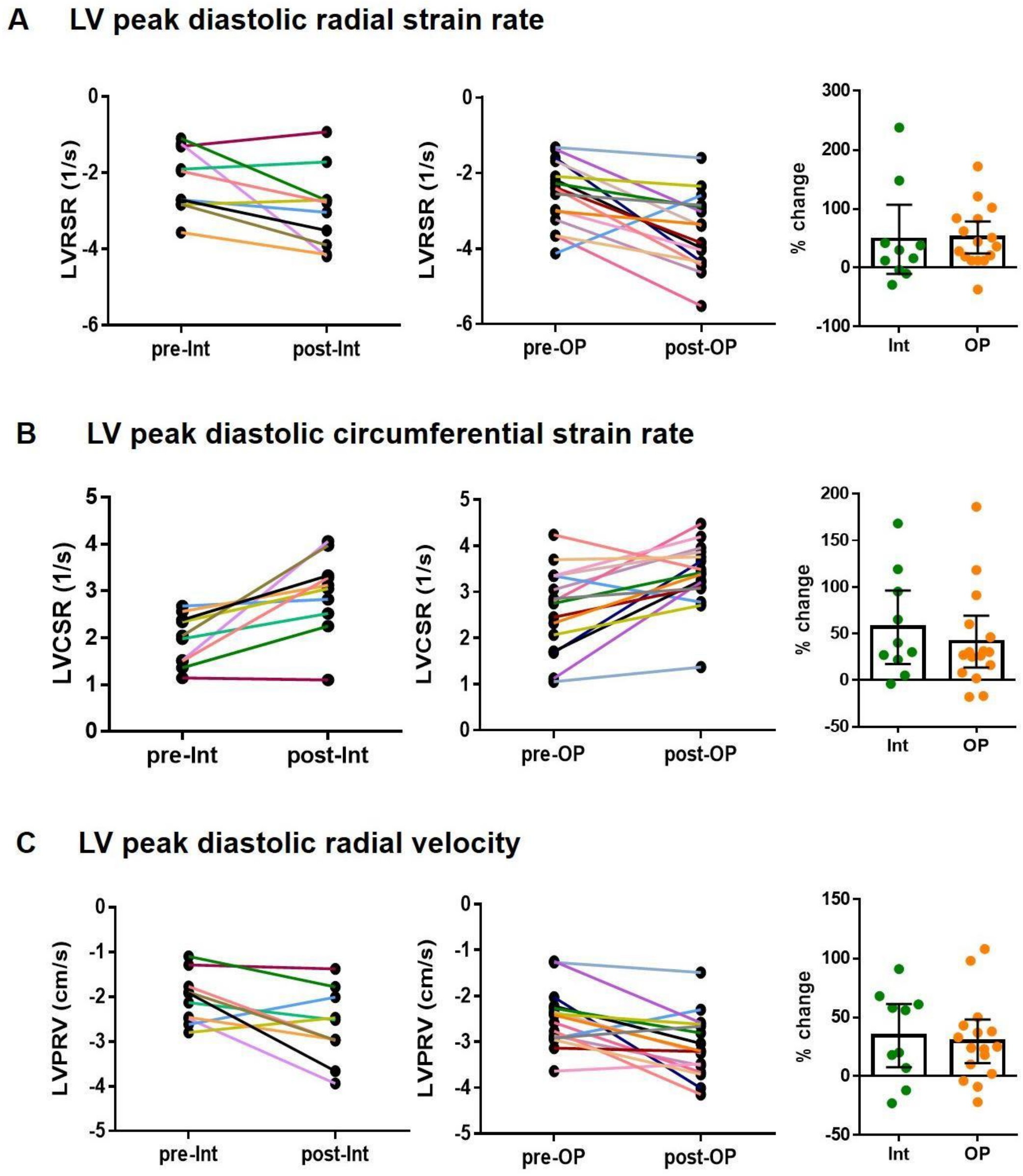

Patients in both cohorts reached similar diastolic strain values post CoA-treatment. The plots illustrate changes in peak diastolic radial (**A**) and circumferential strain rates (**B**) as well as improvement in LV peak diastolic radial velocity (**C**). Graphs in the first and second column illustrate variable progression during hospital stay. The scatter dots plots in the third column illustrate the mean with the 95% confidence interval for the mean. Green dots represent interventional and orange dots represent surgical patients. Abbreviations:LV, left ventricle; LVCSr, LV circumferential strain rate; LVPRD, LV peak radial displacement; LVPRV, LV peak radial velocity; LVRSr, LV radial strain rate; pre-Int, pre Intervention; post-Int, post Intervention; pre-OP, pre-operation; post-OP, post-operation.

REFERENCES

1. Virani SS, Alonso A, Aparicio HJ, Benjamin EJ et al. Heart Disease and Stroke Statistics-2021 Update:A Report From the American Heart Association.*Circulation*.2021;143(8):e254-e743. DOI: 10.1161/CIR.0000000000000950.
2. Rao PS.Coarctation of the aorta.*Curr Cardiol Rep*.2005;7(6):425-434.
3. Carr M, Curtis S, Marek J.EDUCATIONAL SERIES IN CONGENITAL HEART DISEASE: Congenital left-sided heart obstruction.*Echo Res Pract*.2018;5(2):R23-R36.
4. Jashari H, Rydberg A, Ibrahimi P, Bajraktari G, Henein MY.Left ventricular response to pressure afterload in children:aortic stenosis and coarctation:a systematic review of the current evidence.*Int J Cardiol*.2015;178:203-209.
5. Khalil M, Jux C, Rueblinger L, Behrje J, Esmaeili A, Schranz D. Acute therapy of newborns with critical congenital heart disease.*Transl Pediatr*.2019;8(2):114-126.
6. Taksande A, Jameel PZ.Critical Congenital Heart Disease in Neonates:A Review Article. *Curr Pediatr Rev*.2021;17(2):120-126.
7. Vergales JE, Gangemi JJ, Rhueban KS, Lim DS.Coarctation of the aorta - the current state of surgical and transcatheter therapies.*Curr Cardiol Rev*.2013;9(3):211-219
8. Fiore AC, Fischer LK, Schwartz T, et al. Comparison of angioplasty and surgery for neonatal aortic coarctation.*Ann Thorac Surg*.2005;80(5):1659-1665.
9. Sen S, Garg S, Rao SG, Kulkarni S.Native aortic coarctation in neonates and infants: Immediate and midterm outcomes with balloon angioplasty and surgery.*Ann Pediatr Cardiol*. 2018;11(3):261-266.
10. Patel HT, Madani A, Paris YM, Warner KG, Hijazi ZM.Balloon angioplasty of native coarctation of the aorta in infants and neonates: is it worth the hassle?*Pediatr Cardiol*.2001; 22(1):53–57.
11. Früh S, Knirsch W, Dodge-Khatami A, Dave H, Prêtre R, Kretschmar O.Comparison of surgical and interventional therapy of native and recurrent aortic coarctation regarding different age groups during childhood.*Eur J Cardiothorac Surg*.2011;39:898–904. doi:10.1016/j.ejcts.2010.09.048

12. Hartman EM, Groenendijk IM, Heuvelman HM, Roos-Hesselink JW, Takkenberg JJ, Witsenburg M.The effectiveness of stenting of coarctation of the aorta: a systematic review. *EuroIntervention*.2015;11(6):660-668.

13. Fesseha AK EB, Dibardino DJ, Cron SG et al.Neonates with aortic coarctation and cardiogenic shock: presentation and outcomes.*Ann Thorac Surg*.2005;79(5):1650-1655.
